# Monitoring of transfer and internalization of *Escherichia coli* from inoculated knives to fresh cut cucumbers (*Cucumis sativus* L.) using bioluminescence imaging

**DOI:** 10.1038/s41598-021-90584-x

**Published:** 2021-06-01

**Authors:** Yeting Sun, Xiaoyan Zhao, Xiulan Xu, Yue Ma, Hongyang Guan, Hao Liang, Dan Wang

**Affiliations:** 1 Beijing Vegetable Research Center, Beijing Academy of Agriculture and Forestry Sciences, Beijing Key Laboratory of Agricultural Products of Fruits and Vegetables Preservation and Processing, Key Laboratory of Vegetable Postharvest Processing, Ministry of Agriculture and Rural Affairs, Beijing, 100097 China; 2 College of Food Science, Shenyang Agricultural University, Shenyang, 110866 Liaoning China; 3 Longda Food Group Co. LTD, Shandong, 265231 China

**Keywords:** Microbiology, Physiology

## Abstract

Slicing may cause the risk of cross-contamination in cucumber. In this study, knife inoculated with *Escherichia coli* (*E. coli*) was used to cut cucumbers, bioluminescence imaging (BLI) was used to visualize the possible distribution and internalization of *E. coli* during cutting and storage. Results showed that the initial two slices resulted in greater bacterial transfer. The bacterial transfer exhibited a fluctuating decay trend, *E. coli* was most distributed at the initial cutting site. The contaminated area on the surface of cucumber slices decreased during the storage period, which can be attributed to the death and internalization of *E. coli*. The maximum internalization distance of *E. coli* was about 2–3 mm, and did not further spread after 30 min from inoculation. Hence, our results provide useful information for risk management in both home and industrial environment.

## Introduction

Consumers’ demand for fresh-cut vegetable is substantially increasing due to the convenience, freshness and nutritional value^1^. Cross-contamination of microorganisms in a food processing facility and at home is one of the main factors leading to sporadic and epidemic foodborne illness^2^. Fresh-cut cucumbers are a popular convenience food. In the processing of fresh-cut cucumbers, various contaminated contact surfaces may transfer microorganisms to the cucumber slices, eliciting the risk of food cross-contamination^3^. Cutting knives are the major harborage site for bacteria. Once the cucumbers are cut with a contaminated knife, the microorganisms can attach to cut surfaces^4–6^. This contamination poses a great safety risk to fresh-cut cucumbers and threatens people's health. Moreover, cutting inevitably damages the tissues, thereby distrupting the natural protective epidermal barrier^7,8^. This increases the surface humidity and releases intracellular compounds, thus providing favorable conditions for microbial growth^9,10^.

*Escherichia coli* is one of the transferred bacteria associated with cross-contamination of fresh vegetables^11^. *E. coli* can infect stainless steel knife in a short period of time and can be transferred and survived on fresh vegetables during the cutting processing even at a low temperature of 8 °C^12^. Once fresh-cut vegetables are contaminated, it is easy for microorganisms to grow and survive, thereby causing food contamination. Studies have reported that pathogens could be transferred from contaminated knives to lettuce, tomatoes and onions, causing cross-contamination^13–15^. However, different vegetables have different tissue structures and nutrients, different bacterial strains have different transfer and distribution characteristics. The transfer and distribution of different *E. coli* strains on cucumbers may be different. In addition, continuous cutting has an impact on the distribution of strains. Studies reported that when tomatoes are continuously cut with a contaminated knife, the contamination of pathogens would decrease gradually as the number of cuttings increased^16^. However, the distribution of *E. coli* during the continuous slicing of cucumber has not been investigated. There was very little visual evidence and detailed knowledge on the distribution, survival and internalization distance of *E. coli* on cucumber slices during contamination and storage was lacking.

BLI detects bioluminescent bacteria in vivo, thus enabling real-time tracking and monitoring of bacterial attachment and dissemination^17^. This technique was successfully used to track the movement and infection mechanisms of bioluminescent strains in animals or plants^18^. BLI is fast, convenient and non-destructive (no ultrathin section required) compared with plate counting and microscopic observation. Compared to previous research, the purpose of this study was to first apply the BLI technology to the study of food cross-contamination in vivo to real-time monitor the survival of *E. coli* on the surface of cucumber during continuous cutting. Survival and internalization distance of *E. coli* on cucumber slices during storage were also assessed. The results provide safety and risk assessment information for the production of fresh-cut cucumbers.

## Materials and methods

### Material

Cucumber variety “Xiamei 2” (*Cucumis sativus* L.) was purchased from Guoxiangsiyi supermarket in Beijing, China. Cucumbers could be randomly purchased by consumers and were grown in green house of Yifendi Farm in the Changping District, Beijing. They were planted in the soil between the middle of May to the middle of June at 25–32 °C. In September/October (approximately 100 days), mature cucumbers (28–30 cm in size) were sampled and immediately transported to the laboratory after harvest. Storage condition in the distribution chain was room temperature. Cucumbers were cleaned with deionized water, immersed in 0.01% sodium hypochlorite solution (NaClO; pH 6.5) for 2 min, washed three times with sterile water, dried on an aseptic laboratory table at room temperature under incandescent light for 1 h until inoculation. Plants (cucumbers) in this article were cultivated but not wild plants. This article states that the collected plants comply with the IUCN Policy Statement on Research Involving Species at Risk of Extinction.

### Bacterial inoculum preparation

The engineered bioluminescent *E. coli* strain was kindly provided by Beijing Academy of Agriculture and Forestry Sciences. The recombinant plasmid pXX3 that the *lux* operon from *Photorhabdus luminescens* bacteria was obtained by digesting pXen13 with Notl and Xhol then ligated to the similarly digested inverse-PCR product of the transposon vector pKGT452Cβ amplified using the primers pKGT2F/pKGT2R, and carries Cmx resistance gene::*luxCDABE*::Tn*1409*. Plasmid pXX3 was transformed into *E. coli* DH5α, then propagated in the *dam-* and *dcm-*deficient *E. coli* strain ER2925. The strain construction method was described in detail^19^. An aliquot (100 μL) of *E. coli* suspension was cultured in 100 mL of LB broth medium supplemented with chloramphenicol (20 μg/mL) at 37 °C for 48 h to activate the bacteria. The culture was then diluted with sterile phosphate buffered saline (PBS) to obtain the final *E. coli* concentrations of approximately 1, 3, 5, and 7 log CFU/mL.

### Kitchen knife inoculation

A stainless steel kitchen knife (18 cm × 8.2 cm, 1.48 cm tick) was sterilized in 75% ethanol (vol/vol) for 5 min, its surface rinsed with SDW and then dried in cabinet for 30 min. The method refers to Kusumaningrum et al.^20^, with a slight modification. The kitchen knife was respectively dipped in various concentrations of the bacterial suspension for 5 min, following which, the inoculated kitchen knife was placed in a sterile biosafety cabinet to dry for 10 min. A sterile cotton swab was used to scrape the adhered *E. coli* on the knife surface to the 1 mL sterile PBS and then serially diluted (tenfold) in PBS. The diluted droplets (0.1 mL) were then plated on LB agar supplemented with chloramphenicol (20 μg/mL) and incubated at 37 °C for 48 h. The numbers of colonies on the surface of the kitchen knife were counted by the plate count method. The experiment was replicated three times.

### Transfer of *E. coli* on the kitchen knife to cucumber during consecutive cuts

Simulating the force applied during actual cutting, three different concentrations of *E. coli* (3.34 ± 0.21, 5.20 ± 0.13 and 7.06 ± 0.25 log CFU/mL) were used to inoculate the kitchen knife, then cucumber was cut 9 times (1 cm thick per slice) longitudinally by the contaminated knife. Try to use the same force for each cut to reduce the errors. The cucumber slices of same cutting number were placed in a sterile plastic bag together with 100 mL sterile PBS (3 slices per bag). Placed the plastic bag containing cucumber slices in a homogenizer for 3 min to homogenize the sample to obtain the mixed solution, then drew 1 mL of mixed solution and tenfold serially diluted with PBS. The diluted solution (0.1 mL) was plated on LB agar supplemented with chloramphenicol (20 μg/mL) and incubated at 37 °C for 48 h. The number of *E. coli* on cucumber slices was counted by plate count method and logarithmic conversion was performed.

All treatments were replicated three times.

The transfer rate from the kitchen knife to the cucumber slice was determined as^21^:

1 $$ \left( {{\text{CFU}}\;{\text{on}}\;{\text{cucumber}}\;{\text{slice}}/{\text{CFU}}\;{\text{on}}\;{\text{a}}\;{\text{kitchen}}\;{\text{knife}}} \right)\, \times \,100\, = \,{\text{Transfer}}\;{\text{Rate}}\left( \% \right) $$

### Bioluminescence imaging of fresh-cut cucumber slices

#### Distribution and internalization distance of *E. coli* on the cutting surface

Cucumbers were cut transversely into 4 cm-disks using the kitchen knife inoculated with *E. coli* (7 log CFU/mL), then the bioluminescence imaging was performed. Next, each cucumber disk was longitudinally cut into 3 segments by sterile knives. Cucumber sections were then screened for bioluminescence in the dark imaging chamber at room temperature using the high-sensitivity CCD model LB of the in vivo plant imaging system (Night Shade LB 985, Berthold Technologies GmbH & Co.KG, Bad Wildbad, Germany) to monitor the inoculation area and transfer degree of *E. coli*. The imaging parameters for the sensitivity/resolution setup were 4 × 4 binning and a 300 s delay prior to an incremental exposure time of 180 s. Bioluminescent signals were quantified using indiGo software (Berthold Technologies, Oak Ridge, TN, USA), the light intensity was displayed by counts per second (cps).

#### Cross-contamination of consecutive cuts during storage

The kitchen knife was inoculated with *E. coli* to continuously cut the cucumbers into nine disks, which were then stored in sterile polypropylene (PP) boxes (185 mm × 150 mm × 55 mm) at either 4, 10, 25 or 37 °C for 30, 60, 120 or 180 min. Then put the cucumber slices into the imaging room for imaging. The experiment was replicated three times. The longitudinal imaging was to longitudinally cut the cucumber disks. The imaging parameters are the same as 2.5.1.

### Survival of *E. coli* on cucumber slice

Live/dead staining of *E. coli* was performed using propidium iodide (PI) obtained from Solarbio Science and Technology Company (Beijing, China) as described with slight modifications^22^. PI (10 mg) was mixed with 10 mL of sterile PBS (pH 7.4) to prepare the mother liquor with a concentration of 1 mg/mL. The sterile PBS was used to elute the *E. coli* from the cucumber slices contaminated for 2 h, then treated with 100 μL of PI stock solution per milliliter of eluates (final PI concentration was 100 μg/mL). Samples were detected via fluorescence filter cubes B1 (blue) and G1 (green). The live *E. coli* showed green fluorescence under the blue excitation (B1) filter, the PI-labeled dead *E. coli* showed red fluorescence under the green excitation (G1) filter. The Image-Pro plus was used to combine images (Version 6.0.0, Media Cybernetics, Inc.).

### Statistical analysis

Data analyses were performed with one-way analysis of variance using IBM SPSS statistics software 20.0 (IBM Corp., Armonk, NY, USA). Significant differences between groups were determined using Duncan’s test. The level of significance was set at 5% for all analyses. Image processing software were IndiGo software (Berthold Technologies, Oak Ridge, TN, USA) and Image-Pro plus 6.0.0 (Media Cybernetics, Inc.). Data were plotted using OriginPro 8 software (OriginLab Corporation, USA). All methods were carried out according to relevant institutional, national, and international guidelines and legislation.

## Results and discussion

### Evaluation of kitchen knife inoculation

*Concentration of E. coli inoculated on a kitchen knife was shown in* Table 1. When the inoculation concentrations were 1.09 ± 0.08, 3.34 ± 0.21, 5.20 ± 0.13 and 7.06 ± 0.25 log CFU/mL, the amount of *E. coli* on knife were 0.36 ± 0.10, 2.85 ± 0.20, 4.70 ± 0.28 and 6.49 ± 0.01 log CFU/knife. The number of *E. coli* inoculated on the knife increased with the increase of the inoculation level, the number was about 0.5-log less than the inoculation solution. Kusumaningrum et al.^20^ reported that the amount of *Salmonella* Enteritidis and *Staphylococcus aureus* on stainless steel surfaces declined with the decrease of the inoculation concentration, the change pattern was similar to this study. Furthermore, previous reports pointed out that the decay model describing the transfer behavior of pathogenic bacteria during slicing of vegetable was less accurate when using the low inoculum^15^. Thus, the transfer number of *E. coli* on the knife was small under low inoculation concentration, which was not conducive to researching and monitoring the transfer of *E. coli*. In order to reveal *E. coli* transfer, higher inoculation concentrations (3.34 ± 0.21, 5.20 ± 0.13 and 7.06 ± 0.25 log CFU/mL) were selected for subsequent experiments.

**Table 1:** Concentration of *E. coli* inoculated on a kitchen knife.

Inoculum concentration (log CFU/mL)	The concentration of *E. coli* on the knife surface (log CFU/knife)
1.09 ± 0.08^d^	0.36 ± 0.10^d^
3.34 ± 0.21^c^	2.85 ± 0.20^c^
5.20 ± 0.13^b^	4.70 ± 0.28^b^
7.06 ± 0.25^a^	6.49 ± 0.01^a^

Different letters respectively represent statistically significant differences between different inoculation concentrations and infection concentrations (*p* < 0.05).

### *E. coli* transferred from kitchen knife to cucumber

Experimental data showed that the transfer amount and rate of *E. coli* exhibited a fluctuating decay pattern during cutting process (Table 2). It was the highest at the first cut in all treatments, after that data showed a sharp decrease, then slightly increased and leveled off, finally decreased again as the number of slices increased. Moreover, the number of *E. coli* remaining on cucumber slices after 9 cuts was below 100. At low inoculum level (3.34 ± 0.21 log CFU/mL), the recovery amount (slice 1: 0.95 ± 0.10; slice 2: 0.68 ± 0.14; slice 3: 0.31 ± 0.14 log CFU/cucumber slice) and transfer rate (slice 1: 1.259; slice 2: 0.676; slice 3: 0.288%) of *E. coli* on the first 3 slices decreased significantly as the cutting number increased. Then they increased significantly at the fourth slice (recovery amount: 0.65 ± 0.11 log CFU/cucumber slice; transfer rate: 0.631%) and the changes on the following data points became stable. When the ninth slice was reached, the recovery amount (0.26 ± 0.16 log CFU/cucumber slice) and transfer rate (0.257%) of *E. coli* were decreased rapidly. This was similar to the previous study, the bacterial transfer that occurs after cutting food with a blade inoculated with 10^4^ CFU/mL bacterial solution showed a fluctuating decrease trend^23^. At moderate and high inoculation levels (5.20 ± 0.13 log CFU/mL and 7.06 ± 0.25 log CFU/mL), higher amount and transfer rate of *E. coli* appeared (3.36 ± 0.03 and 3.75 ± 0.08 log CFU/cucumber slice; 4.571% and 0.182%). The number of *E. coli* recovered from the fourth slice under moderate-concentration and fifth slice of cucumber under high-concentration were reduced by approximately 2.8 and 4.05 log CFU/cucumber slices, respectively, were about 3.3 and 4.62 log CFU lower than the inoculum level on the knife. The amount of *E. coli* transferred on the next slice tended to be flat, then slowly decreased. The transfer of bacteria depends on the nature of the contaminated surface, cutting speed, force and cutting action^24,25^. Stainless steel is a hydrophilic surface, which can be used as a medium for bacteria to attach, may promote the release of pathogens during the food preparation process and relocate them to the surface of high-moisture food^26^. The cutting speed and action were kept as consistent as possible in this study, all cutting action were the longitudinal cutting that perpendicular to the cutting board, therefore cutting speed and action might have little effect on the transfer of *E. coli*. However, the force was variable during the cutting process, the fluctuating transfer of *E. coli* might be affected by force^7^. The similar trends were observed by other researchers with regard to other pathogenic bacteria that transfer through surface of stainless steel to cucumbers, lettuce and celery^20,27,28^. Furthermore, in previous researches, the transfer behavior of pathogenic bacteria during the slicing process of fish, meat and vegetables were similar which could use the decay model to describe^29–31^. The transfer of *E. coli* was probably because the shearing force on the knife surface, which was not determined in our study, might have affected the removal of loosely attached cells, resulting in the transfer of bacterial cells on the knife to the cucumber slices^32^. As the slice continued, the number of cells transferred back to the knife gradually decreased and cells transferred to cucumer slice with the next cut gradually decreased, this might be because the knife was simultaneously affected by adhesion and hydrophobicity. When the attachment between *E. coli* and knife was not as strong as *E. coli* and cucumber, the cells might detachment from the knife and movement to cucumber slice so that transfer becomes more feasible. The remain of exudate released from the cucumber slices altered the hydrophobicity of the knife surface and affected the transfer of *E. coli*. This caused some transferred cells to move back onto the knife^33^. Furthermore, the dual- or multiple- species of endophytes in cucumber exudate might affect the adhesion of *E. coli* and stainless steel^34^. Changes in the transfer rate during the continuous cutting process, which might be because consecutive cutting was not a static, neither an easy-to-control process^21^.

**Table 2:** The transfer of *E. coli* from the kitchen knife to the cucumber surface.

Inoculum concentration (log CFU/mL)	Concentration of *E. coli* on knife (log CFU/knife)	number of cuts	The concentration of *E. coli* on the cucumber surface (log CFU/cucumber slice)	Transfer rate (%)
3.34 ± 0.21	2.85 ± 0.20	1	0.95 ± 0.10^a^	1.259
		2	0.68 ± 0.14^bc^	0.676
		3	0.31 ± 0.14^de^	0.288
		4	0.65 ± 0.11^bc^	0.631
		5	0.85 ± 0.21^ab^	1.000
		6	0.70 ± 0.00^abc^	0.708
		7	0.53 ± 0.17^cd^	0.479
		8	0.75 ± 0.12^abc^	0.794
		9	0.26 ± 0.16^e^	0.257
5.20 ± 0.13	4.70 ± 0.28	1	3.36 ± 0.03^a^	4.571
		2	3.17 ± 0.02^b^	2.951
		3	2.20 ± 0.15^c^	0.316
		4	1.90 ± 0.08^d^	0.158
		5	1.82 ± 0.13^d^	0.132
		6	1.94 ± 0.14^d^	0.174
		7	1.85 ± 0.05^d^	0.141
		8	1.48 ± 0.08^e^	0.060
		9	1.36 ± 0.14^e^	0.046
7.06 ± 0.25	6.49 ± 0.01	1	3.75 ± 0.08^a^	0.182
		2	3.30 ± 0.09^b^	0.065
		3	2.94 ± 0.05^c^	0.028
		4	2.70 ± 0.05^d^	0.016
		5	2.44 ± 0.09^e^	0.009
		6	2.58 ± 0.07^de^	0.012
		7	2.02 ± 0.06^f^	0.003
		8	1.78 ± 0.17^g^	0.002
		9	1.82 ± 0.02^g^	0.002

Different letters in the same column indicate statistically significant differences (*p* < 0.05).

### BL images of *E. coli* transfer and distribution on cucumber slice after cutting

**Figure 1:**
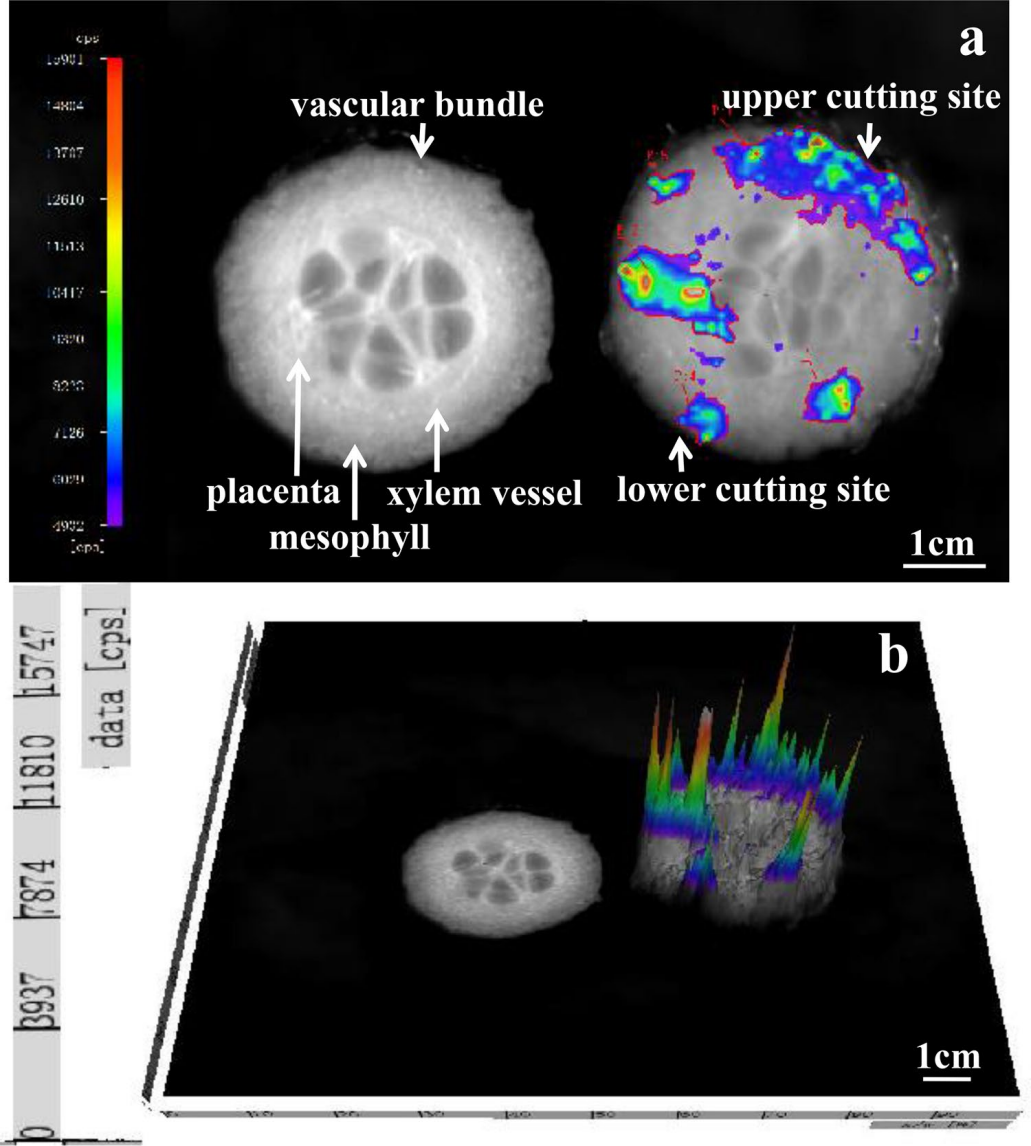
Immediate BL images of *E. coli* transfered and distributed on the cross-section of cucumber. (**a**) The left side was the control of uncontaminated cucumber slices, the right side was the cucumber slice after cutting horizontally by a knife inoculated with *E. coli* (7.06 ± 0.25 log CFU/mL). (**b**) 3D surface chart of the same processed sample. The red color represented a strong signal and the blue color represented a weak signal. IndiGo software (https://www.nchsoftware.com/accounting/index.html?kw=sage%20software&gclid=AIaIQobChMI3MvFj-zr7wIVj2gqCh14fAmLEAEYASAAEgJPqPD_BwE, https://softwaretopic.informer.com/).

The high inoculation (7.06 ± 0.25 log CFU/mL) was used to reveal the transfer of *E. coli* during the cross-contamination process according to previous research^33^. BL images of *E. coli* transferred and distributed on the cross-section of cucumber were shown in Fig. 1a. Our results suggested that the adhesion of the initial cleavage site was strongest due to mechanical action, most *E. coli* transferred to the upper section. The cells transferred back to the knife due to the hydration of the exudate, *E. coli* would be randomly distributed on the tissues in the middle and lower parts of the cucumber slices. The *E. coli* luminescence signal detected on the vascular bundles, xylem vessel and placental tissues was the strongest at the site of inoculation, representing the most transfer of strains. This might be because these tissues have xylem tissues that could transport nutrients and water to cucumbers^12^, which provided nutrients for growth of *E. coli*. These tissues would accumulate and adhesion of *E. coli* more easily than other tissues and the detected luminescent signal will be stronger than other tissues. The 3D surface chart further showed that more luminescence signals were detected at the initial cleavage site and stronger luminescence signals were detected in the xylem tissues (Fig. 1b).

### Survival of *E. coli* on cucumber slices during storage

**Figure 2:**
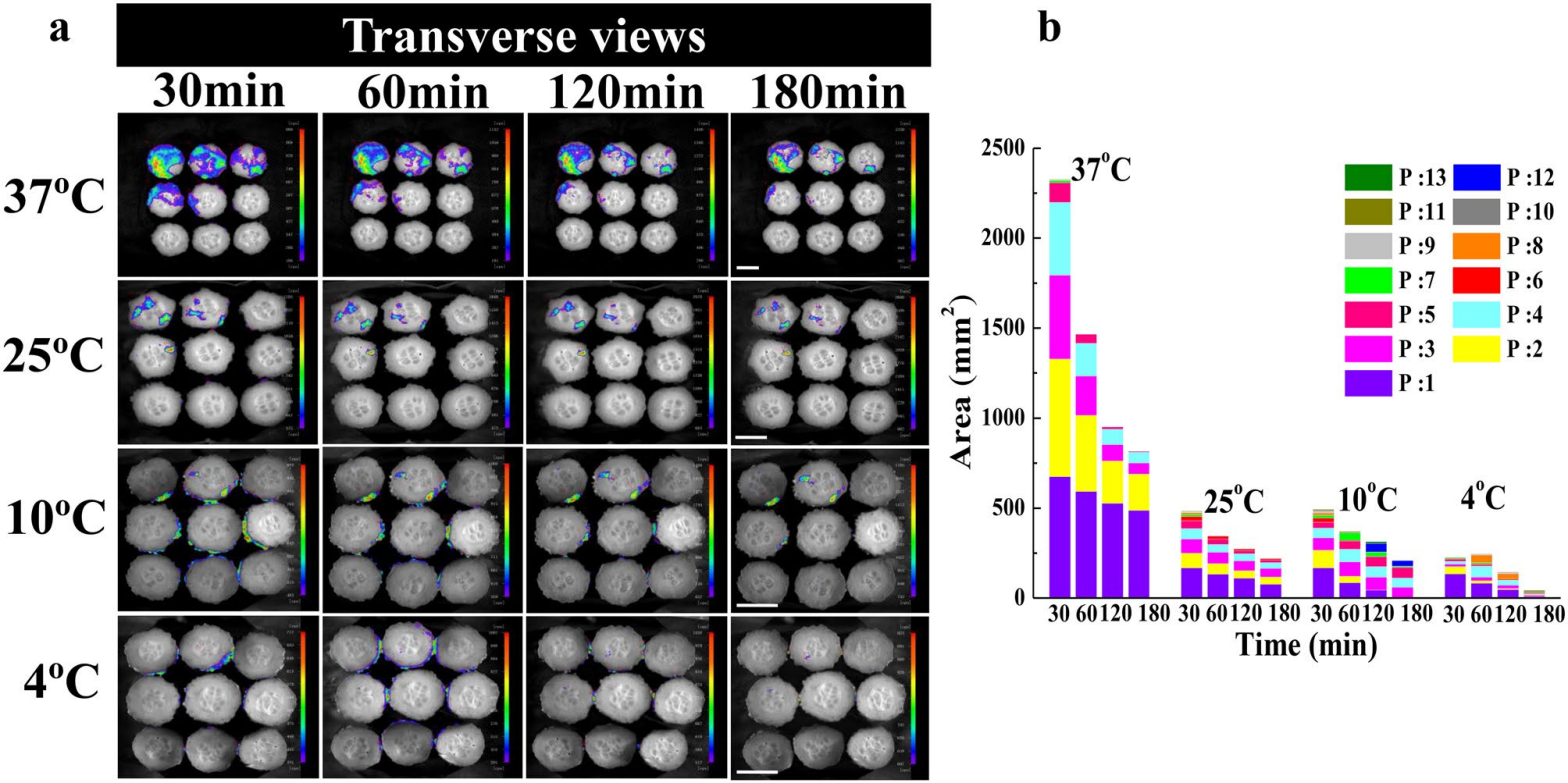
BL images of the transfer and distributed of *E. coli* on the cucumber slices under different storage temperatures and times. (**a**) The first row of cucumber slices from left to right represented cut numbers of 1, 2, 3; the second row represented 4, 5, 6; the third row represented 7, 8, 9. The scales are the same under the same temperature. All scales represent 3 cm. (**b**) The bar graph showed the contaminated area of *E. coli* on cucumber slices after cutting under each storage condition. Different contaminated areas were indicated by different colors. IndiGo software (https://www.nchsoftware.com/accounting/index.html?kw=sage%20software&gclid=AIaIQobChMI3MvFj-zr7wIVj2gqCh14fAmLEAEYASAAEgJPqPD_BwE, https://softwaretopic.informer.com/). OriginPro 8 software (https://en.freedownloadmanager.org/users-choice/Origin_8_Free_Download_Full_Version.html, https://softwaretopic.informer.com/).

**Figure 3:**
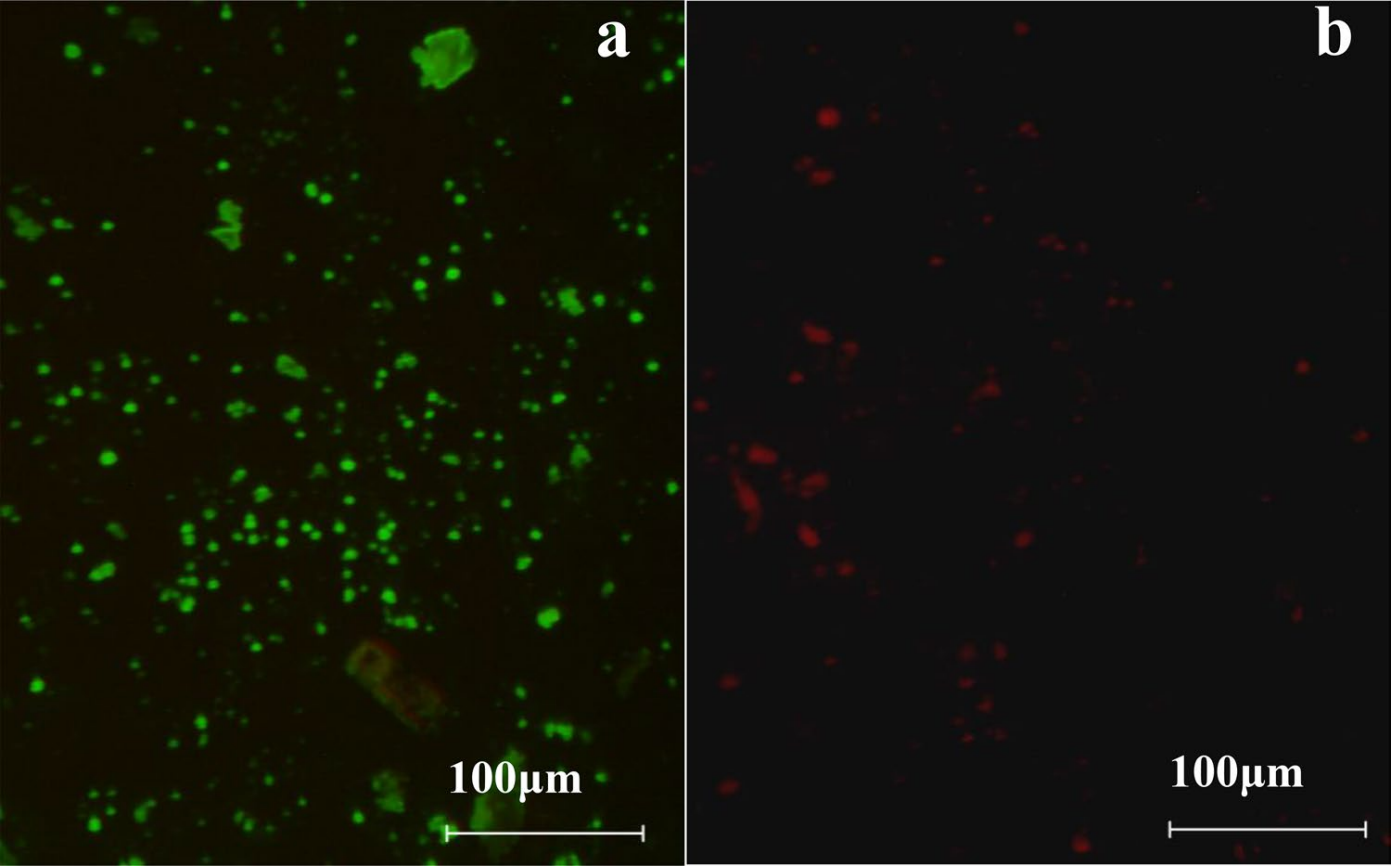
Fluorescence microscopy images of *E. coli* (× 100). Fluorescence microscopy image of live/dead bacteria on cucumber slices inoculated with *E. coli* after 30 min (**a**) and 2 h (**b**) from inoculation. Image-Pro plus 6.0.0 (https://www.xrayscan.com/software-image-pro-plus/, https://www.xrayscan.com/).

BL images showed that the contaminated area of *E. coli* on all cucumber slice samples gradually decreased with the decrease of storage temperature and the extension of storage time. More specifically, the luminescent signal of the *E. coli* under 37 °C storage condition was strongest (Fig. 2). This might be because 37 °C was the suitable temperature for the growth of *E. coli*. *E. coli* was irregularly distributed on vascular bundles, xylem vessels, placenta and mesophyll tissues where the signal color were the reddest and the signal were the strongest. The survival of *E. coli* on cucumber slice after cutting 5 times was significantly reduced, the luminescent signal of *E. coli* was below the minimum detection limit. Compared with other storage temperatures, the distribution area of *E. coli* under 37 °C at the edge decreased sharply as the storage time. The reduction of contaminated area and the weakening of the bioluminescent signals might be caused by *E. coli* death and internalization. The death of *E. coli* was verified by the fluorescence microscope images in Fig. 3. It was found that more number of living *E. coli* were observed on the cucumber slices in the early stage of inoculation 30 min after cross-contamination (Fig. 3a), while more *E. coli* deaths were observed after 2 h of inoculation (Fig. 3b). The death of *E. coli* might be due to the gradual consumption of water and nutrients on the surface of cucumber slices with the extension of storage time. This might also be caused by the release of hydrogen peroxide from damaged plant tissue that causes oxidative stress in the bacterial cells and the presence of competing microorganisms^20^, thereby harming or inactivating *E. coli*^35^. However, the antibacterial hydrogen peroxide produced by the wound will temporarily affect the attachment of *E. coli* and tends to decrease after injury^36^, so some bacteria will survive^37^. Under other temperature conditions (25 °C, 10 °C, 4 °C), *E. coli* mainly colonized and distributed on the margins, placenta and vascular system tissues. However, the attenuation degree of *E. coli* in the same contaminated area was much lower than 37 °C with the storage time. These temperatures (25 °C, 10 °C, 4 °C) are not suitable for the growth of *E. coli*, leading to differences in the growth and attenuation metabolism of *E. coli* on cucumbers. However, previous studies have shown that pathogenic bacteria could survive at 4 °C and grow at 10 °C, indicating that unintentional abuse of temperature could cause the growth of pathogenic bacteria, enough to reach potentially hazardous levels^38^. The survival ability of *E. coli* was stable due to the decreased ability of self-protection and regulation of plant cells during low temperature storage of cucumber^39^. The increased activity of POD and SOD could kill the superoxide free radical produced in adversity^40^, reduce the damage to *E. coli*. Meanwhile, the exudate released from the cucumber slices provided sufficient nutrition for the survival of the bacteria and maintained their activity. These results showed that there were still potential safety hazards in cutting cucumbers with contaminated knives even under low temperature conditions, the distribution of *E. coli* tended to be on the initial cutting site and nutrient-rich tissues. In addition, it was observed that the transfer area and change trend of *E. coli* after cutting cucumber using a kitchen knife inoculated with 10^5^ and 10^3^ CFU/mL of *E. coli* during storage was similar to the concentration of 10^7^ CFU/mL (data not shown).

### BLI of *E. coli* internalization during storage

**Figure 4:**
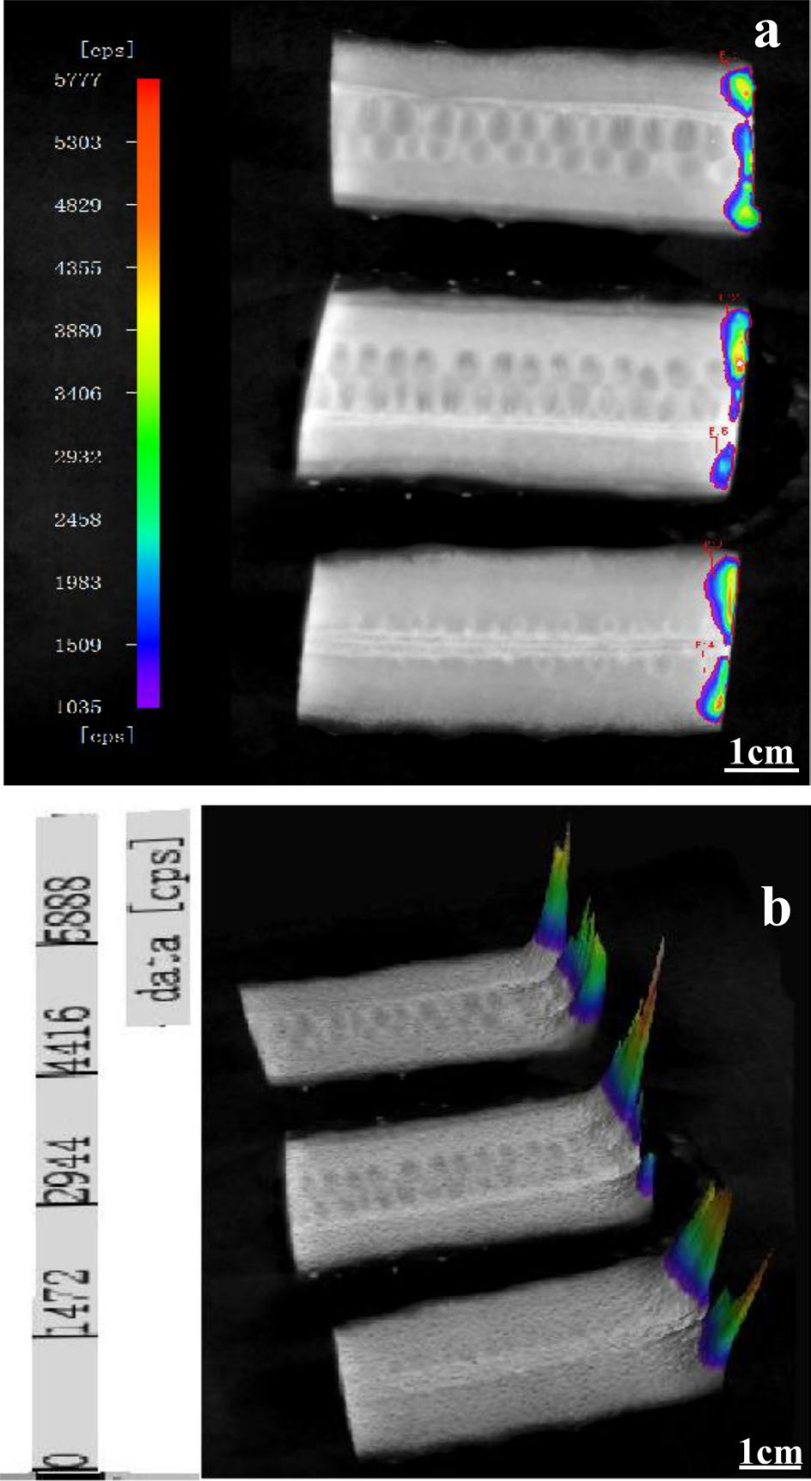
BL images of longitudinal section of *E. coli* internalized from the cross-section of cucumber. (**a**) Bacterial internalization image of cutting cucumber horizontally (cut surface of cucumber is on the right, 7.06 ± 0.25 log CFU/mL) with a bacterial knife and then cutting longitudinally. (**b**) The 3D surface chart of the same processed sample. The red color represented a strong signal and the blue color represented a weak signal. IndiGo software (https://www.nchsoftware.com/accounting/index.html?kw=sage%20software&gclid=AIaIQobChMI3MvFj-zr7wIVj2gqCh14fAmLEAEYASAAEgJPqPD_BwE, https://softwaretopic.informer.com/).

**Figure 5:**
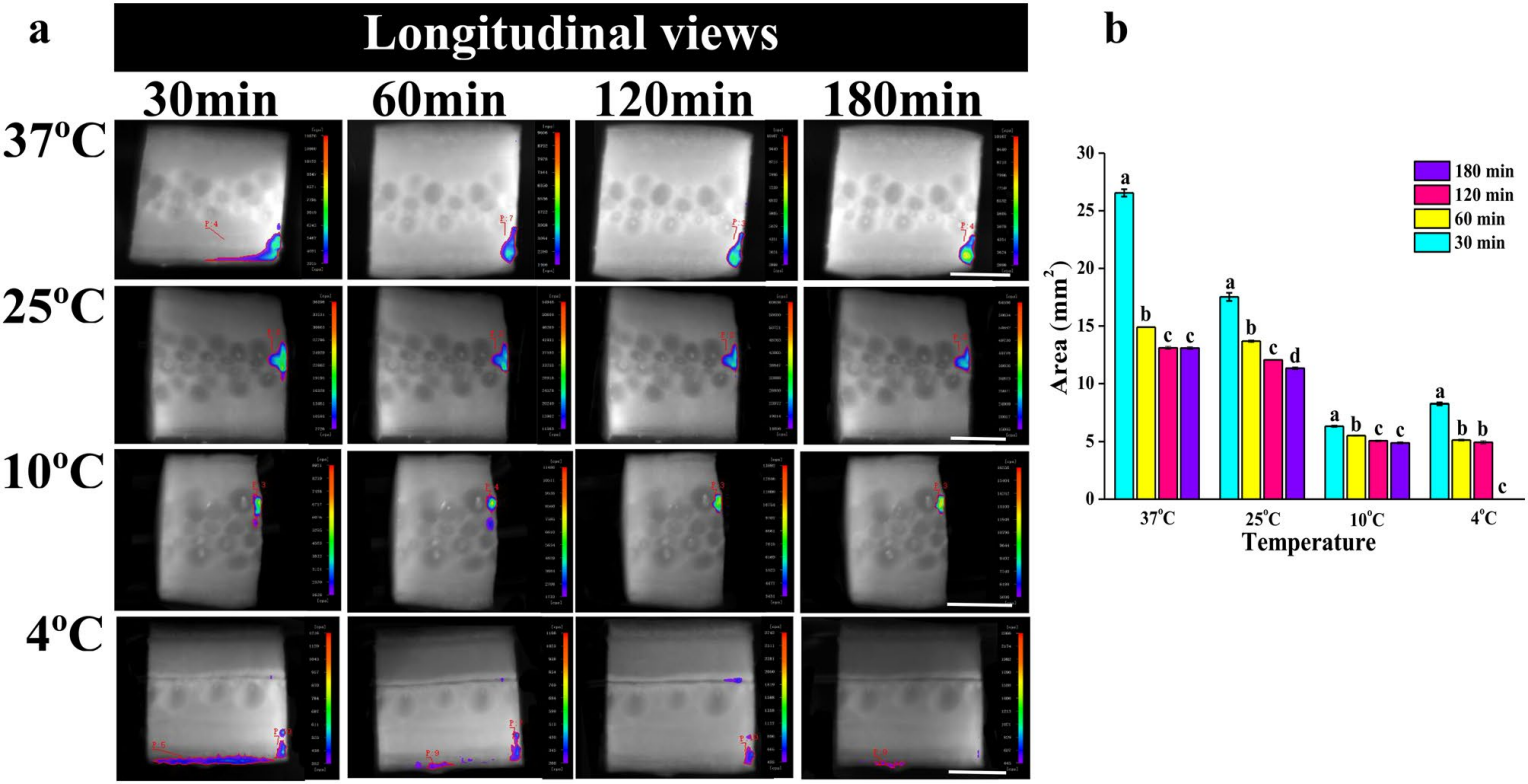
BL images of *E. coli* internalization on the longitudinal section of cucumber under different storage temperatures and times. (**a**) The first row of cucumber slices from left to right represented cut numbers of 1, 2, 3; the second row represented 4, 5, 6; the third row represented 7, 8, 9. The scales are the same under the same temperature. All scales represent 1 cm. (**b**) The bar graph showed the contaminated area of *E. coli* internalized on the longitudinally cut cucumber slices under each storage condition. Different contaminated areas were indicated by different colors. Different letters represent statistically significant differences (*p* < 0.05). IndiGo software (https://www.nchsoftware.com/accounting/index.html?kw=sage%20software&gclid=AIaIQobChMI3MvFj-zr7wIVj2gqCh14fAmLEAEYASAAEgJPqPD_BwE, https://softwaretopic.informer.com/). OriginPro 8 software (https://en.freedownloadmanager.org/users-choice/Origin_8_Free_Download_Full_Version.html, https://softwaretopic.informer.com/).

*E. coli* was internalized from the attachment point of cucumber, then, spread to the surrounding tissues (Fig. 4a). The maximum internalization distance was 2–3 mm after 30 min from inoculation. The 3D surface chart further clarified that the bioluminescent signals were the strongest on the inoculated surface, and became weaker with an increase in internal distance (Fig. 4b). Regarding the internalization distance, the result obtained in the present study was in agreement with previous research findings^41^ indicating that microorganisms could penetrate into the medium, the penetration distance was in the first 2–3 mm of the medium surface, rendering the bacteria more difficult to detect. The internalization of *E. coli* at different storage times and temperatures was shown in Fig. 5. The internalized area of all samples decreased rapidly with storage time, being constant stable after 60 min from inoculation. This might be due to the temporarily affect the growth of *E. coli* by cucumber exudate, which led to a rapid decrease in internalized *E. coli*. After a period of storage, the bacterial cells had adapted to the growth environment and could survive then stabilized. *E. coli* reached the maximum internalization distance after 30 min from inoculation at 37 °C without change with the extension of storage time. The internalization distance of *E. coli* declined with temperature decreased, and remained unchanged after 30 min. Less *E. coli* internalization distance and area was observed at refrigeration temperatures (4 and 10 °C), but the viability of *E. coli* was weaker at 4 °C. The internalization is probably due to the fact that vascular bundles, xylem vessels function in transporting nutrients and water, thus providing access to *E. coli* to the internal tissues^42,43^. But the refrigeration temperatures were not suitable for bacterial growth, they could reduce the activity of *E. coli*. Results further proved that the decreased of the contaminated area on the cucumber slice and the luminescence signal were caused by the internalization of *E. coli* as well as the death of *E. coli*.

## Conclusion

This study determined that the concentration of *E. coli* contaminated on the surface of the kitchen knife was around 0.5 log lower than the inoculation concentration. Consecutive cutting of cucumber slices with a contaminated knife would cause cross-contamination, which was a dynamic process. The maximum transfer area of *E. coli* was at the initial cutting site of cucumber slices. Once *E. coli* adhered to the vascular bundles and xylem tissues, it would spread along these tissues into the internal tissues of cucumber and the maximum internalization distance was 2–3 mm. The contaminated area of *E. coli* on cucumber slices gradually decreased with storage time, which might be due to the death and internalization of *E. coli*. Quantifying and visualizing the relevant cross-contamination during cucumber cutting validated the transfer and internalization of bacterial and could provide a scientific basis for risk management strategies to reduce, prevent or eliminate cross-contamination in the kitchen as well as in the industrial environment.
